# Intraplatelet L-Arginine-Nitric Oxide Metabolic Pathway: From Discovery to Clinical Implications in Prevention and Treatment of Cardiovascular Disorders

**DOI:** 10.1155/2020/1015908

**Published:** 2020-03-03

**Authors:** Jakub Gawrys, Damian Gajecki, Ewa Szahidewicz-Krupska, Adrian Doroszko

**Affiliations:** Department of Internal Medicine, Hypertension and Clinical Oncology, Wroclaw Medical University, Poland

## Abstract

Despite the development of new drugs and other therapeutic strategies, cardiovascular disease (CVD) remains still the major cause of morbidity and mortality in the world population. A lot of research, performed mostly in the last three decades, revealed an important correlation between “classical” demographic and biochemical risk factors for CVD, (i.e., hypercholesterolemia, hyperhomocysteinemia, smoking, renal failure, aging, diabetes, and hypertension) with endothelial dysfunction associated directly with the nitric oxide deficiency. The discovery of nitric oxide and its recognition as an endothelial-derived relaxing factor was a breakthrough in understanding the pathophysiology and development of cardiovascular system disorders. The nitric oxide synthesis pathway and its regulation and association with cardiovascular risk factors were a common subject for research during the last decades. As nitric oxide synthase, especially its endothelial isoform, which plays a crucial role in the regulation of NO bioavailability, inhibiting its function results in the increase in the cardiovascular risk pattern. Among agents altering the production of nitric oxide, asymmetric dimethylarginine—the competitive inhibitor of NOS—appears to be the most important. In this review paper, we summarize the role of L-arginine-nitric oxide pathway in cardiovascular disorders with the focus on intraplatelet metabolism.

## 1. Introduction

After establishing the real nature of EDRF by Furchgott et al. [1, 2], which appeared to be nitric oxide (NO), numerous other groups were working on the nitric oxide synthesis pathway and its potential role in human (patho)physiology. This led to the discovery of the nitric oxide synthase [3] which produces nitric oxide from L-arginine with flavin adenine dinucleotide (FAD), flavin mononucleotide (FMN), tetrahydrobiopterin (BH4), and heme with a zinc atom as cofactors. From that time, numerous functions of NO were established which can generally be divided into three groups:

1. Group associated with neuronal transmission, where the NO plays an inhibitory role as a mediator in peripheral nonadrenergic noncholinergic (NANC) neurotransmission (causing relaxation mainly in the gastrointestinal tract, penile corpus cavernosum, and bladder) [4]
2. Group playing an inflammatory role, where NO is produced by the inducible isoform of nitric oxide synthase (iNOS)
3. Group related to the cardiovascular system

## 2. Nitric Oxide in Cardiovascular Disorders

Despite the development of new drugs and other therapeutic strategies, cardiovascular disease (CVD) remains still the major cause of morbidity and mortality in the world population [5]. A lot of research, performed mostly in the last three decades, revealed an important correlation between “classical” demographic and biochemical risk factors for CVD (i.e., hypercholesterolemia [6], hyperhomocysteinemia [7], smoking [8], renal failure [9], aging [10], diabetes [11], and hypertension [12]) with endothelial dysfunction associated directly with the nitric oxide deficiency. In the vascular endothelium, NO is produced by the endothelial isoform of nitric oxide synthase (eNOS = NOS3) which is constitutively active, allowing the maintenance of appropriate vascular tone by constant vasodilating action [13]. The other functions of NO are inhibition of platelet aggregation, inhibition of smooth muscle proliferation, and leucocyte interaction with the vascular wall [14]. All of these properties place nitric oxide as a key modulator of vascular homeostasis. Nowadays, endothelial dysfunction, defined as a reduction in the endothelial NO bioavailability, can be measured noninvasively by the change in blood flow (e.g., EndoPAT 2000 and brachial flow-mediated dilation) or appropriate agonists (e.g., reaction to acetylcholine administered by iontophoresis measured by laser Doppler flowmetry) [15]. There are several mechanisms which can limit the bioavailability of NO. One of them is a decrease in the eNOS expression in endothelial cells which occurs in advanced atherosclerosis [16] and in smokers [17]. Decreased NO production can also be an effect of L-arginine deficiency or nitric oxide synthase cofactors. A lot of studies have been performed on the oxidative stress as a factor limiting the NO bioavailability [18]. An imbalance between the creation of reactive oxygen species (ROS) and their scavenging by antioxidants promotes the reaction between NO and O2^−^ which results in the peroxynitrite formation. Peroxynitrite is a potent oxidative compound which promotes posttranslational modifications of proteins (including the eNOS protein) [19], alterations in the main metabolic pathways [20], or eNOS uncoupling which results in the production of superoxide anion instead of NO [21, 22]. Increased formation of peroxynitrite and other reactive oxygen species has been demonstrated in established cardiovascular system disorders [23] and is associated with a vast majority of CVD risk factors such as hypertension [24], diabetes [25], tobacco use [26], and hypercholesterolemia [27]. Another mechanism responsible for nitric oxide deficiency, which is deeply investigated, is connected with competitive inhibition of nitric oxide synthase by asymmetric dimethylarginine (ADMA)—a naturally occurring amino acid circulating in plasma and present in various tissues and cells.

## 3. ADMA as the Most Potent Inhibitor of the L-Arginine-Nitric Oxide Pathway

The first mention about asymmetric dimethylarginine presence comes from the study by Kakimoto and Akazawa who have isolated its crystalline form, among other substances, by ion-exchange chromatography of the aliphatic basic amino acid fraction of human urine [28]. By the fact that its concentration in urine is not affected by arginine administered orally, the authors assumed that this compound may be a derivate from endogenous protein proteolysis. In 1992, Leone et al. proposed its potential pathophysiological role by providing *in vitro* and *in vivo* evidence that ADMA inhibits NO synthesis [29]. In addition, they described the accumulation of dimethylarginines by the lack of urine production in patients with end-stage chronic renal failure as a potential mechanism of hypertension and immune dysfunction in this group of patients.

**Figure 1:**
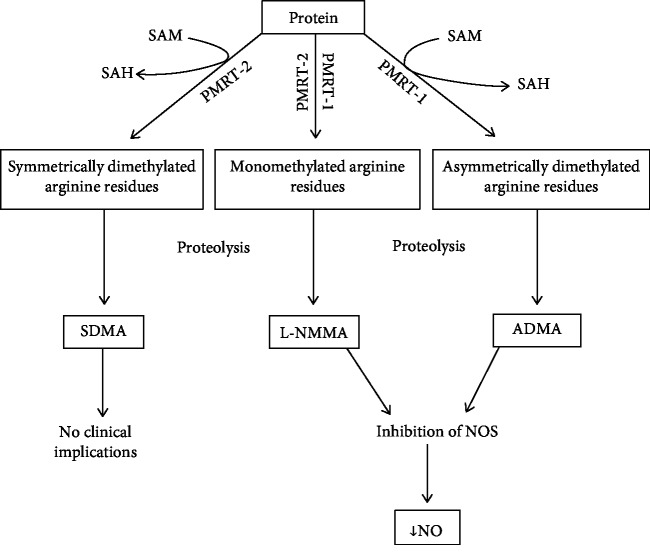
Synthesis of ADMA from methylated proteins. SAM: S-adenosylmethionine; SAH: S-adenosylhomocysteine; PMRT-1: protein methyltransferase-1; PMRT-2: protein methyltransferase-2; SDMA: symmetric dimethylarginine; L-NMMA: monomethylated arginine; ADMA: asymmetric dimethylarginine; NOS: nitric oxide synthase; NO: nitric oxide. Based on [30–32].

Methylated derivates of arginine are produced as a result of proteolysis of endogenous methylated proteins, i.e., histones. This methylation is catalysed by two isoforms of the arginine methyltransferases (PMRTs)—PMRT-1 and PMRT-2 proteins—with S-adenosylmethionine as a donor of methyl residues. As an effect of PMRT-1—the main isoform present in the vascular wall (endothelial and smooth muscle cells)—asymmetrically dimethylated and monomethylated arginine residues are formed. PMRT-2 is also capable of mono- and dimethylation of arginine residues, but in this case, residues are dimethylated symmetrically [30, 32]. After protein degradation, methylarginine compounds appear at the beginning in the cytosol but also in plasma [31]. Monomethylated arginine (L-NMMA) and asymmetric dimethylarginine (ADMA) are inhibitors of all nitric oxide synthase isoforms whereas symmetric dimethylarginine (SDMA) is not (Figure 1). The inhibitory effect of ADMA and L-NMMA on NOS is similar [33], but considering that plasma concentration of ADMA is up to tenfold higher than that of L-NMMA, ADMA was an object of research for the last decades. The inhibition of NOS may not be the only effect of asymmetric dimethylarginine in human. There are reports that at high concentrations, both ADMA and SDMA may compete in the transport through the Y-amino acid transporter with arginine [34] and also may inhibit the Na+/K+ ATPase [35]. However, concentrations required for these actions seem to be too high to be clinically relevant. Murray-Rust et al. proposed another potential target for ADMA, which is the arginine-glycine amidinotransferase. The structure of this enzyme is similar to that of dimethylarginine dimethylaminohydrolases (DDAHs) that metabolize ADMA [36]. However, Vallance and Leiper suggest that ADMA is only a poor inhibitor of this transferase [37].

**Figure 2:**
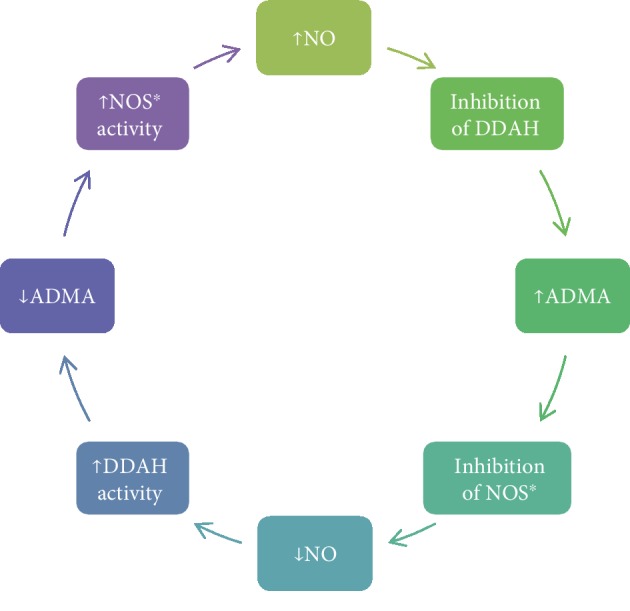
Potential homeostatic mechanism of autoregulation of nitric oxide production. NO: nitric oxide; NOS: nitric oxide synthase; ADMA: asymmetric dimethylarginine; DDAH: dimethylarginine dimethylaminohydrolase; ∗: reactions involving inducible nitric oxide synthase. Based on [38, 39].

All of the methylated arginine derivates are eliminated by kidneys. In contrast to SDMA, which is excreted completely by kidneys, ADMA and L-NMMA are also degraded by DDAH [38, 40]. As a result, citrulline and the monomethylamines are formed. The catalytic site of DDAH involves cysteine residue. Its nitrosylation by reactive nitrogen species renders the enzyme inactive which can be the potential homeostatic mechanism especially in reactions involving the inducible NOS (iNOS) (increased production of NO leads to the accumulation of ADMA by inhibiting the DDAH) [39] (Figure 2). In addition, this cysteine residue is susceptible to the action of the number of oxidative stress-related cardiovascular risk factors such as hypercholesterolemia, hypertension, renal failure [41], hyperhomocysteinemia, hyperglycaemia [42], and tobacco smoking [43], which also results in the accumulation of ADMA. This can be a pathway, allowing different factors to affect endothelial function [44]. There are two known isoforms of DDAH. DDAH-1 accompanies the neuronal NOS and is present in the liver, kidneys, and lungs (its action contributes to circulating ADMA concentration), while DDAH-2 is present in tissues expressing endothelial and inducible NOS and dominates in vessels, especially endothelium and smooth muscle cells (its role is connected with the located regulation of the amount of ADMA) [38, 45].

Concentrations of ADMA in plasma, which are connected with biologic action, are approximately 10-fold higher than concentrations observed under physiological condition. It suggests that even if plasma concentration of ADMA indicates its amount in the whole body, it does not mean that the same concentrations are present in all tissues [31, 46]. In the study by Cardounel et al., where the inhibition of NO generation by bovine endothelial cells was measured, the effect of raising ADMA concentrations was greater than expected on the basis of kinetic studies [47]. It suggests that there should be a mechanism of methylarginine uptake by cells, which, according to Bogle et al., could be the y^+^ transport system [34]. The regulation of transport may be the explanation for “L-arginine paradox.” This term is used to refer situations where exogenous administration of L-arginine led to enhancement of endothelial vasodilatory function, which is present despite the fact that its plasma concentration is up to 30-fold higher than necessary to completely saturate the NOS [48]. In physiological conditions, plasma level of ADMA is insufficient to compete with L-arginine in transport through the endothelial cell membrane [49]. However, in subjects with developed CVD or with a high CVD risk profile, elevated ADMA concentrations are able to affect the eNOS activity. The restoration of eNOS activity and improvement of vasodilatory function of the endothelium by the addition of exogenous L-arginine in pathological conditions, without effects in healthy subjects, point to the L-arginine/ADMA ratio, instead of L-arginine and ADMA concentrations alone, as the main factor regulating the NO bioavailability [50, 51].

## 4. The Role of ADMA in Cardiovascular Disease Development

After the discovery of ADMA and the establishment of its function in the L-arginine → nitric oxide → cGMP pathway, the research focused on the connection of elevated ADMA concentrations with CVD and classic cardiovascular risk factors. One of the first studies evaluating the role of ADMA was performed by Bode-Böger et al. They showed that elevated concentrations of asymmetric dimethylarginine are found in hypercholesterolemic rabbits and it is the first biochemical abnormality observed at the early stage of atherosclerosis [52]. The following studies led to the discovery that elevated ADMA plasma concentrations are present in humans with hypercholesterolemia and with vascular disease. This finding was associated with endothelial dysfunction and impairment in the NO production measured by lower excretion of nitrates in the urine and worse NO-dependent forearm vasodilation [53]. It led to the conclusion that elevated ADMA concentration is an early marker of endothelial dysfunction known as a prognostic marker of severe cardiovascular events. At the end of the previous century, Cardillo et al. discovered that hypertension is associated with a defect in NO synthesis [54]. As a result, impaired endothelium-dependent vasodilation occurred, but the reaction for isoproterenol and sodium nitroprusside, which both enhance the NO concentration, was preserved. It means that endothelial dysfunction in hypertension is an effect of the selective decrease in NO bioavailability. Other studies proved that in early stages, hypertension is connected with the elevated plasma level of ADMA. Sonmez et al. compared the population of healthy adults with a demographically matched group of people with a recent diagnosis of hypertension, yet without any medical intervention [55]. Their work indicates that even in the initial stage of the disease, hypertensive patients have a higher level of plasma ADMA concentrations when other factors such as age, BMI, smoking history, CRP level, total cholesterol, LDL cholesterol, and triglyceride levels were similar between the two groups. Other studies on the link between ADMA and hypertension are in line with the previous results [56]. In addition, Curgunlu et al. demonstrated that ADMA concentration is elevated not only in hypertensive subjects but also in individuals with white coat hypertension, which indicates endothelial dysfunction presence in this state. Numerical values of ADMA concentrations place people with white coat hypertension in the continuum between normotensive (lower) and hypertensive (higher ADMA concentrations) subjects [57].

Aging is one of the main risk factors for cardiovascular disease and is connected with the progression of endothelial dysfunction. This resulted in the hypothesis that aging may be associated with increased ADMA concentrations. Gates et al. compared ADMA levels in a group of young adults (18-26 years old) with those in older ones (52-71 years old) without accompanying diseases except for impaired endothelial function measured by forearm flow-mediated dilation. The lack of difference in ADMA concentrations between these groups points to another reason for the dysfunction of endothelium during aging other than competitive inhibition of NOS [58].

One of the main risk factors for coronary artery disease and other cardiovascular disorders is tobacco smoking. Sobczak et al. investigated the influence of active and passive smoking for ADMA concentration in healthy volunteers without other CVD risk factors. The ADMA concentrations were higher in active and passive smokers when compared to the nonsmoking control group. However, these differences were not statistically significant [59]. Other research on the effect of tobacco smoking on the NO bioavailability presented similar results, but most of them were performed on a population with already developed cardiovascular disease [60, 61]. Despite the fact that some authors observed higher ADMA plasma concentrations in healthy people smoking >20 cigarettes daily [62], it seems that endothelial dysfunction and higher CVD risk related to tobacco use are not connected with alterations in NO bioavailability.

In contrast to smoking, hyperhomocysteinemia is among the risk factors that probably cause endothelial dysfunction by elevating plasma levels of asymmetric dimethylarginine. There are some hypotheses regarding the exact pathway in which concentrations of these compounds are connected. The first of them is that hyperhomocysteinemia causes increased methylation of proteins when ADMA is a product of proteolysis [63]. This hypothesis was initially supported by detecting higher ADMA concentrations after the hyperhomocysteinemic meal in humans [64]. However, there are other possible mechanisms such as stimulating SAM-dependent activity of PRMTs, decreased renal excretion, or inhibiting the DDAHs—enzymes responsible for ADMA degradation. Supporting the last hypothesis, Stühlinger et al. found that higher ADMA concentrations after exposure to homocysteine or methionine are connected with decreased activity of the DDAH isoforms [42]. The same authors observed direct inhibition of recombinant human DDAH activity by homocysteine in cell-free systems. What is more, other studies showed that mice with hyperhomocysteinemia had decreased levels of mRNA for the two major DDAH enzymes [65]. Considering the above-given reports, further research is needed to establish the exact mechanism of ADMA concentration elevation by homocysteine.

**Figure 3:**
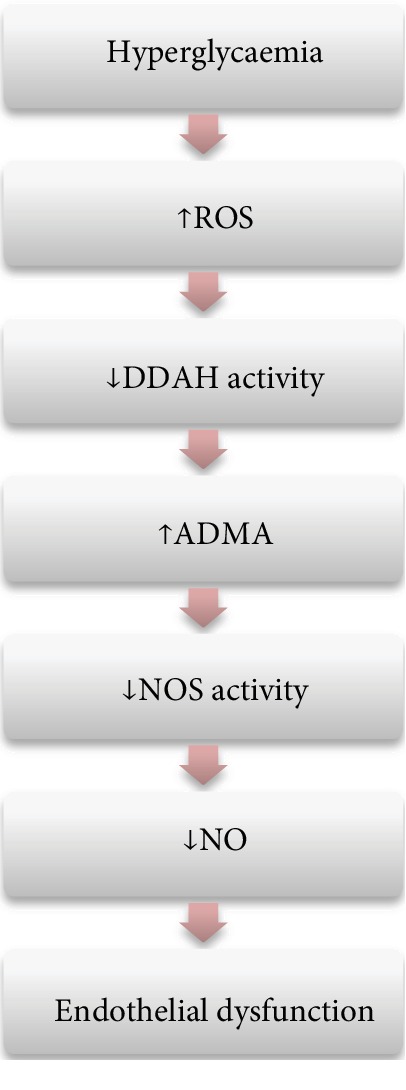
The effect of hyperglycaemia on the L-arginine-nitric oxide pathway. ROS: reactive oxygen species; DDAH: dimethylarginine dimethylaminohydrolase; ADMA: asymmetric dimethylarginine; NOS: nitric oxide synthase; NO: nitric oxide. Authors' modification based on [66–69].

Metabolic disorders such as obesity, insulin resistance, and diabetes mellitus (DM) are known to be the risk factors for cardiovascular disease development. In all of them, endothelial dysfunction appears to play a pivotal role. It has been proposed that elevated levels of plasma ADMA concentration are responsible for the impairment in the NO bioavailability in metabolic disorders (Figure 3). Almost every diabetic person (with the exception of young subjects with type 1 diabetes) is considered a patient with a high CVD risk profile. In this population, even with no atherosclerosis and other organ damage, elevated ADMA levels are obtained [66]. The ADMA concentrations rise not only in general but also in dynamic situations. Fard et al. investigated changes in ADMA level 5 hours after ingestion of a high-fat meal. Their study demonstrated acute elevation of its concentration followed by a decreased vasodilatory response of the brachial artery measured by flow-mediated dilation which can be another factor promoting the development of atherosclerosis [67]. Higher ADMA level is a predictor of poor prognosis in patients with DM and already developed coronary artery disease [68] and is also a predictor of acute cardiovascular events in DM patients without vascular changes [69]. Endothelial dysfunction connected with elevated ADMA concentration is present also in prediabetic states such as obesity and insulin resistance [70–72] and is considered the first step in the development of atherosclerosis. The intensity of its dysfunction is higher in insulin-resistant subjects than in obese but insulin-sensitive ones [73]. Some research has shown that weight loss (provided by bariatric surgery followed by diet or only by diet), as well as reduction of insulin resistance (by pharmacological and nonpharmacological treatment), resulted in lowering of plasma ADMA levels and in the improvement of endothelial function [71, 73]. Searching for an explanation of elevated ADMA concentrations in metabolic disorders, Lin et al. conducted a study in which the possible pathways of ADMA accumulation in diabetic rats were investigated [74]. The discovery was that this accumulation is connected with reduced endothelial DDAH activity. However, the amount of DDAH found in the aortic endothelium of both groups was comparable. This suggests that these effects are reversible which is consistent with the studies mentioned above. As DDAH is sensitive to oxidative stress [36], hyperglycaemia-induced release of free radicals may be responsible for the elevation of ADMA concentration and endothelial dysfunction in metabolic disorders.

## 5. NOS Pathway in Platelets (Figure 4)

Shortly after the discovery of the L-arginine-nitric oxide pathway, the presence of nitric oxide synthase activity in human platelets was reported by Radomski et al. [75]. The tests performed on the specially prepared platelet cytosol showed that an increase in cGMP concentration was shown not only with direct NO donors (sodium nitroprusside) but also with L-arginine, known as a substrate of nitric oxide synthase. The effect of L-arginine is dependent on the presence of NADPH which indicates the enzymatic character of this reaction. The formation of NO in the platelet cytosol was inhibited by the addition of competitive NOS inhibitors such as L-NMMA which provides evidence of the presence of the nitric oxide synthesis pathway in human platelets. The L-arginine addition to the platelet cytosol did not increase the basal level of cGMP when platelets were not activated by collagen. It shows that the exogenous substrate, such as L-arginine, can be used by platelet NOS only after activation which probably potentiates the transport of the substrate through the platelet membrane [76]. However, the presence of nitric oxide synthase and all NO pathway components was questioned by some authors. Subjects to doubt were, among others, contamination of the probes with another blood cells [77], lack of specificity of used assays [78], measurement of cGMP activity or the amount of L-citrulline as indicators of NOS activity without considering other metabolic pathways [79, 80], or measurement of inorganic NO metabolites [81]. Finally, Cozzi et al. directly visualized the nitric oxide production by collagen-induced platelets using an NO-specific fluorescent agent—4-amino-5-methylamino-2′,7′-difluorofluorescein diacetate (DAF-FM) [82]. This agent reacts with NO and provides a fluorescent signal. The specificity of this compound was tested by incubation in an NO donor and H_2_O_2_—fluorescence occurred only in solution with an NO donor. What is more, in tests conducted on platelets from eNOS-knockout mice, fluorescence was not observed—it further confirms the specificity of DAF-FM. The results of this study confirmed the presence of eNOS in platelets by the increase in DAF fluorescence in platelets adhering to type I collagen under flow. The intensification of the signal was dependent on the presence of the NOS substrate (L-arginine), as well as of the competitive NOS inhibitor (L-NMMA).

Despite the low concentrations of nitric oxide produced in platelets (compared to the endothelial cells) [82], it appears to have an important role in the aggregation of thrombocytes. According to Tymvios et al., platelet aggregation is regulated by endogenous NO. Inhibiting of all the endogenous NO action resulted in fatal thromboembolism in mice, but the deletion of the eNOS gene did not affect platelet reactivity [84]. This suggests that other sources of NO production, identified to be platelet-derived, are responsible for the regulation of aggregation and recruitment of thrombocytes. The increase in the amount of platelet-derived nitric oxide (PDNO) works as a negative feedback mechanism limiting the number of recruited thrombocytes during thrombus formation [75]. Impaired platelet NO production results in intensified surface P-selectin expression, which promotes coagulation by enhancing the expression of the tissue factor [85]. Considering the fact that platelet-derived NO release upon activation is delayed, its role is more complex. It not only limits the process of aggregation but also allows the recruitment of the required amount of cells to form the hemostatic clot [86]. Alterations of this subtle mechanism can play a crucial role in the development of cardiovascular diseases. Several studies provided data that impaired platelet-derived nitric oxide availability is connected with the intensity of coronary disease risk factors. Ikeda et al. showed the inverse correlation of PDNO with age, mean arterial pressure, hypercholesterolemia, and smoking [87]. What is more, the decrease in PDNO production was also correlated with a number of risk factors present in each individual. The impaired platelet-derived nitric oxide release is present also in already developed cardiovascular disorders such as coronary artery disease [88]. LDL cholesterol reduces L-arginine transport into platelets which is followed by reduction of the NO production [89]. It has been shown that statins have the potential to restore the PDNO release, which results in an improvement in the regulation of platelet aggregation [90, 91]. Platelet NOS activity is also impaired in subjects suffering from diabetes, both types 1 and 2 [92].

Patients with type 2 diabetes are characterized by impaired production of NO and cGMP with no changes in the amount of nitric oxide synthase. Intraplatelet level of cGMP presents an inverse correlation with glycated haemoglobin and blood glucose levels [93]. It suggests that impairment in platelet-derived NO production in this population may be associated with glycaemia-dependent suppression of platelet NOS activation [83].

Intraplatelet NO signalling is also affected in essential hypertension. Some studies show decreased platelet-derived NO release [94] and downregulation of receptors (ỳ^+^ transport system) responsible for membrane L-arginine transport [95]. The inhibition of L-arginine transport, according to Brunini et al., is connected with elevated levels of ADMA and L-NMMA [96]. What is more, the use of specific agonists for NOS3 with different pathways of action did not result in an increase in its activity in hypertensive subjects [97]. It indicates the enzyme defect as the main reason for the impairment of platelet NO release. Although plasma ADMA concentrations are elevated in a hypertensive subject compared to a healthy subject, Tymvios with colleagues demonstrated that elevated plasma ADMA level does not alter platelet NO production [84]. It suggests the existence of another mechanism controlling PDNO release. On the contrary, De Meirelles et al. found that plasma ADMA and L-NMMA are capable of decreasing the intraplatelet cGMP concentration which corresponds with lower NOS activity [98]. Previously cited, Cozzi et al. [82] showed an impact of the competitive NOS inhibitor—L-NMMA—on the release of nitric oxide by platelets. There is a possibility that disruptions of the NO synthesis process in the abovementioned situations are the effect of the accumulation of NOS inhibitors in thrombocytes. Further research is necessary to test this hypothesis and evaluate its clinical importance.

Recent studies have shown another interesting aspect regarding PDNO release and its potential role in the hemostasis and thrombus generation. The discovery of two subpopulations of platelets, with and without the presence of intraplatelet eNOS, allowed the determination of a new hypothesis on a thrombus generation mechanism. In response to vascular injury, eNOS^neg^ platelets (about 20% of all thrombocytes) adhere to the damaged area. This process is facilitated by the lack of endogenous NO production by this subpopulation. eNOS^neg^ platelets, by the secretion of metalloproteinase-2, recruit eNOS^pos^ ones (80% of the thrombocyte population), which, by their higher COX-1 content and higher thromboxane production, form the majority of the emerging aggregate. However, their ability to produce NO results in the limitation of the thrombus size [99] [100]. In vitro studies showed that increase in the eNOS^neg^/eNOS^pos^ ratio, as well as inhibition of eNOS, promotes platelet aggregation. Changes in this ratio may be responsible for the impairment of blood coagulation homeostasis and may predispose individuals to developing CVD. It has been shown that platelets from patients after acute coronary syndrome produce less NO when compared to those from healthy ones [88]. Further research is needed to fully understand and determine the role of alterations in platelet subpopulations or their potential function as a target for new therapeutic strategies.

Little is known about the effect of antiplatelet drugs on NO release by thrombocytes. Inhibition of the GPIIb/IIIa receptor (responsible for fibrinogen binding during platelet aggregation) resulted in the enhancement of NO production and reduction of the formation of superoxide anion [101]. Acetylsalicylic acid (ASA) has different effects on NOS activity dependent on dose-dependent mechanisms of action and duration of the treatment. On the one hand, ASA reduces NOS activity by limiting the NOS-activating response to stimulation of platelet beta-adrenergic receptors—this effect is shared with other nonsteroidal anti-inflammatory drugs so it appears to be mediated through COX inhibition. On the other hand, acute in vivo and in vitro action of aspirin results in the acetylation of the platelet NOS and thereby in COX-independent activation of this enzyme. Of clinical relevance, chronic administration of small doses of ASA (75 mg per day) did not enhance platelet NOS activity in a COX-independent mechanism, but the response to beta-adrenergic stimulation remains reduced [102, 103]. What is more, Rothwell et al. showed that an optimal dose of ASA depends on bodyweight and that for subjects above 70 kg, a daily dose of 75 mg is insufficient to reduce cardiovascular events properly [104]. It suggests that the methodology of already conducted studies should be carefully revised.

## 6. Conclusions

The knowledge regarding the exact pathogenesis of impaired production of platelet-derived nitric oxide may have important clinical implications. Cardiovascular disorders are frequently related to enhanced thrombus formation. What is more, several conditions, despite the proper antiplatelet treatment, are associated with an elevated incidence of cardiovascular events. Identification of the patients with higher risk, for example, by assessment of platelet-derived nitric oxide production impairment or changes in the eNOS^neg^/eNOS^pos^ ratio, may enable the application of the more appropriate, individualized treatment or early implementation of proper prevention. More research on the exact relation between cardiovascular disorders and the amount of nitric oxide synthesized by platelets is necessary to fully determine their clinical importance. Finally, the knowledge about the biochemistry and exact pathways of PDNO actions may serve as a basis for creating new or using already known drugs in new indications.

## Figures and Tables

**Figure 4:**
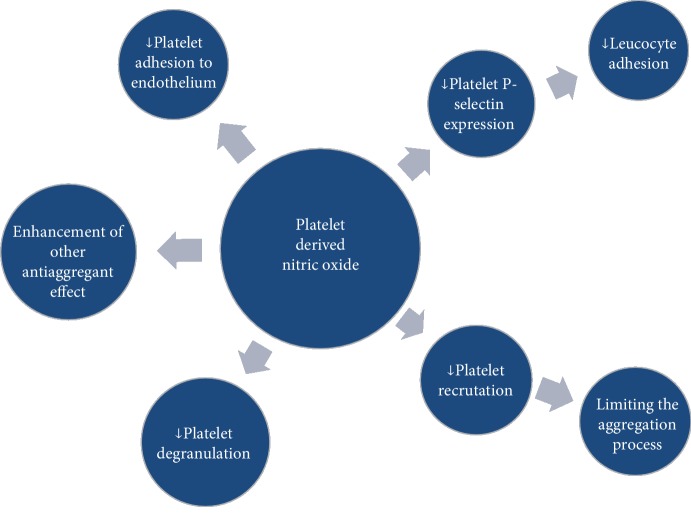
The known functions of platelet-derived nitric oxide. Authors' modification on the basis of [83].
